# Can Therapeutic Exercise Slow Down Progressive Functional Decline in Patients With Amyotrophic Lateral Sclerosis? A Meta-Analysis

**DOI:** 10.3389/fneur.2020.00853

**Published:** 2020-08-13

**Authors:** Donghwi Park, Sang Gyu Kwak, Jin-Sung Park, Yoo Jin Choo, Min Cheol Chang

**Affiliations:** ^1^Department of Physical Medicine and Rehabilitation, Ulsan University Hospital, University of Ulsan College of Medicine, Ulsan, South Korea; ^2^Department of Medical Statistics, College of Medicine, Catholic University of Daegu, Daegu, South Korea; ^3^Department of Neurology, School of Medicine, Kyungpook National University, Kyungpook National University Chilgok Hospital, Daegu, South Korea; ^4^Department of Rehabilitation Medicine, College of Medicine, Yeoungnam University, Daegu, South Korea

**Keywords:** amyotrophic lateral sclerosis, therapeutic exercise, conventional exercise, motor function, respiratory function, meta-analysis

## Abstract

**Background:** Despite the high incidence of muscle weakness in patients with amyotrophic lateral sclerosis (ALS), the effects of therapeutic exercise on these individuals have not been clearly determined.

**Methods:** A comprehensive database search was conducted on PubMed, Embase, Cochrane Library, and SCOPUS. We included studies published up to December 31, 2019 that fulfilled our inclusion and exclusion criteria. Functional status was determined as the Amyotrophic Lateral Sclerosis Functional Rating Scale (ALSFRS) score (previous and revised versions) before and after a therapeutic exercise program for the meta-analysis. The Cochrane Collaboration's tool for assessing risk of bias in randomized trials was used for the methodological quality assessments of included studies. The meta-analysis was performed using the RevMan v.5.3.

**Results:** A total of 94 patients in the experimental group (who received therapeutic exercise) and 159 patients in the control group (who received conventional exercise or therapy) were included from five randomized controlled trials. The decrement of ALSFRS (previous version), ALSFRS-R (revised version), and ALSFRS-R-Respiratory scores at the 6-month evaluation were less for the therapeutic exercise group as compared to the control group. However, at the 6-month evaluation, the ALSFRS-R-Limb scores did not significantly differ between the two groups.

**Conclusions:** Therapeutic exercise appears beneficial for patients with ALS. Further, it appears to exert more of a cardiopulmonary benefit, as opposed to preventing the progression of limb weakness. However, as the therapeutic exercises applied in each included study were not uniform, the result of our meta-analysis should be considered cautiously.

## Introduction

Amyotrophic lateral sclerosis (ALS) is characterized by rapidly progressive degeneration of motor neurons in the primary motor cortex, brainstem, and spinal cord (1, 2). This degeneration leads to progressive muscle atrophy and weakness (3). Patients with ALS usually succumb to respiratory complications, on average, within 5 years of symptom onset (1, 4–7). Worldwide, the annual incidence rates for ALS are reportedly 0.4–2.4 cases per 100,000 (8–11). The incidence increases with each decade of life until at least the seventh decade. The prevalence rates of ALS are 4–10 cases per 100,000 (8–11).

Muscle weakness is considered one of the major symptoms of ALS. In more than 70% of patients, the presenting symptom is muscle weakness in the focal upper or lower extremities (12). Initial muscle weakness usually occurs in isolated muscles, most often distally, and is followed by rapidly progressive weakness and functional limitations. Although a few agents can inhibit the progression of symptoms, ALS has no cure (1). Therefore, patients with ALS must maintain activities of daily living as much as possible with symptomatic treatments such as therapeutic exercises (13, 14). These exercises consist of strengthening, resistive, or active aerobic exercises (13, 14). However, there are very few randomized, controlled, large-scale studies that have evaluated the potential benefits of therapeutic exercise in patients with ALS (15, 16).

The role of therapeutic exercise in patients with ALS has been controversial, and the possibility of causing work-related damage secondary to excessive exercise or strengthening exercises is a concern (15). For example, highly repetitive or heavy resistance exercise can cause prolonged loss of muscle strength in weakened or denervated muscle. In addition, some previous epidemiologic data showed a higher incidence of ALS in individuals who performed intense work, or who regularly engaged in high levels of physical activity before disease onset (17). This has reinforced clinicians to caution against exercise for patients with ALS.

In any case, the marked reduction in physical activity that often accompanies ALS can lead to cardiovascular deconditioning and muscle weakness secondary to disuse (18). These weaknesses are superimposed on the weakness caused by the disease itself. Reduced physical activity, particularly if prolonged, also produces muscle atrophy, osteoporosis, and reduced strength of tendons and ligaments. Some previous studies have attempted to report the efficacy of several therapeutic exercises on functional deterioration and quality of life in patients with ALS (18–21). For example, performance-based activities such as walking, standing, and moderate-load resistance strength training reportedly have some beneficial effect on physical functions in patients with ALS. However, the effects of therapeutic exercise in patients with ALS have not been clearly elucidated because of the small number of previous studies. To further explore this issue, we performed a meta-analysis of all available clinical studies of therapeutic exercise treatments in patients with ALS.

## Materials and Methods

### Search Strategy

This meta-analysis was performed according to the Preferred Reporting Items for Systematic Reviews and Meta-Analysis (PRISMA) guidelines. We systematically searched the relevant literature contained in PubMed, Embase, Cochrane Library, SCOPUS, CINAHL, and LILACS for studies published up to December 31, 2019. The following keywords were used for the database search: (amyotrophic lateral sclerosis AND exercise). The filters were used to select studies with human participants. Also, a manual search of the reference lists of the published articles was conducted to identify additional eligible studies for review.

### Eligibility Criteria

We applied the following inclusion criteria for selection of articles: (1) included patients were diagnosed with ALS or probable ALS; (2) therapeutic exercise (strengthening, resistive, or active aerobic exercises) was conducted, (3) patients' functional states were evaluated both before and after the therapeutic exercise; (4) the study design was a randomized or quasi-randomized controlled trial. We excluded review articles, letters, and case reports. We also excluded studies when the study reported no data/results, or the reported data/results were insufficient. In addition, observational studies were not included.

### Study Selection and Data Extraction

After discarding duplicated studies, two reviewers (DP and MCC) independently evaluated the potentially eligible studies. Articles were screened for eligibility based on a review of the title and abstract, and disagreements were resolved through consensus. After screening, the full texts of eligible articles were read independently by the two reviewers, and the eligibility of each article was re-assessed. Subsequently, the data, including first author, publication date, number of patients, demographic information (age, sex, and disease duration), exercise mode, loss to follow-up, adverse effect, and outcome data, were extracted. Our primary outcome measures included the scores of ALS Functional Rating Scale (ALSFRS)-Total, ALSFRS-R-Total, ALSFRS-R-Respiratory, and ALSFRS-R-Limb.

### Quality Assessment

To determine the methodological quality of the included studies, the Cochrane Collaboration's tool for assessing risk of bias in randomized trials was used to determine adequate sequence generation, allocation concealment, blinding, incomplete outcome data, selective outcome reporting, and other potential sources of bias. The judgments of bias were expressed as “low risk,” “high risk,” or “unclear risk” (22).

### Statistical Analysis

RevMan v.5.3 software (http://tech.cochrane.org/revman) was used for statistical analysis of the pooled data. Heterogeneity tests are performed on each analysis to measure the degree of discrepancy between the results (23). Heterogeneity across studies was assessed using the Cochran's Q test. *P*-values of <0.05 were considered to have substantial heterogeneity and a random effect model was used for data analysis. In contrast, when *p*-value was above 0.05, the pooled data was considered homogeneous and a fixed effect model was applied. We analyzed the standardized mean difference (SMD), which is the difference of scores measured in the experimental (who received therapeutic exercise) and control groups (who received conventional exercise or therapy). Further, the 95% confidence interval (CI) was used in the analysis. *P*-value < 0.05 was considered statistically significant.

## Results

### Study Selection

**Figure 1:**
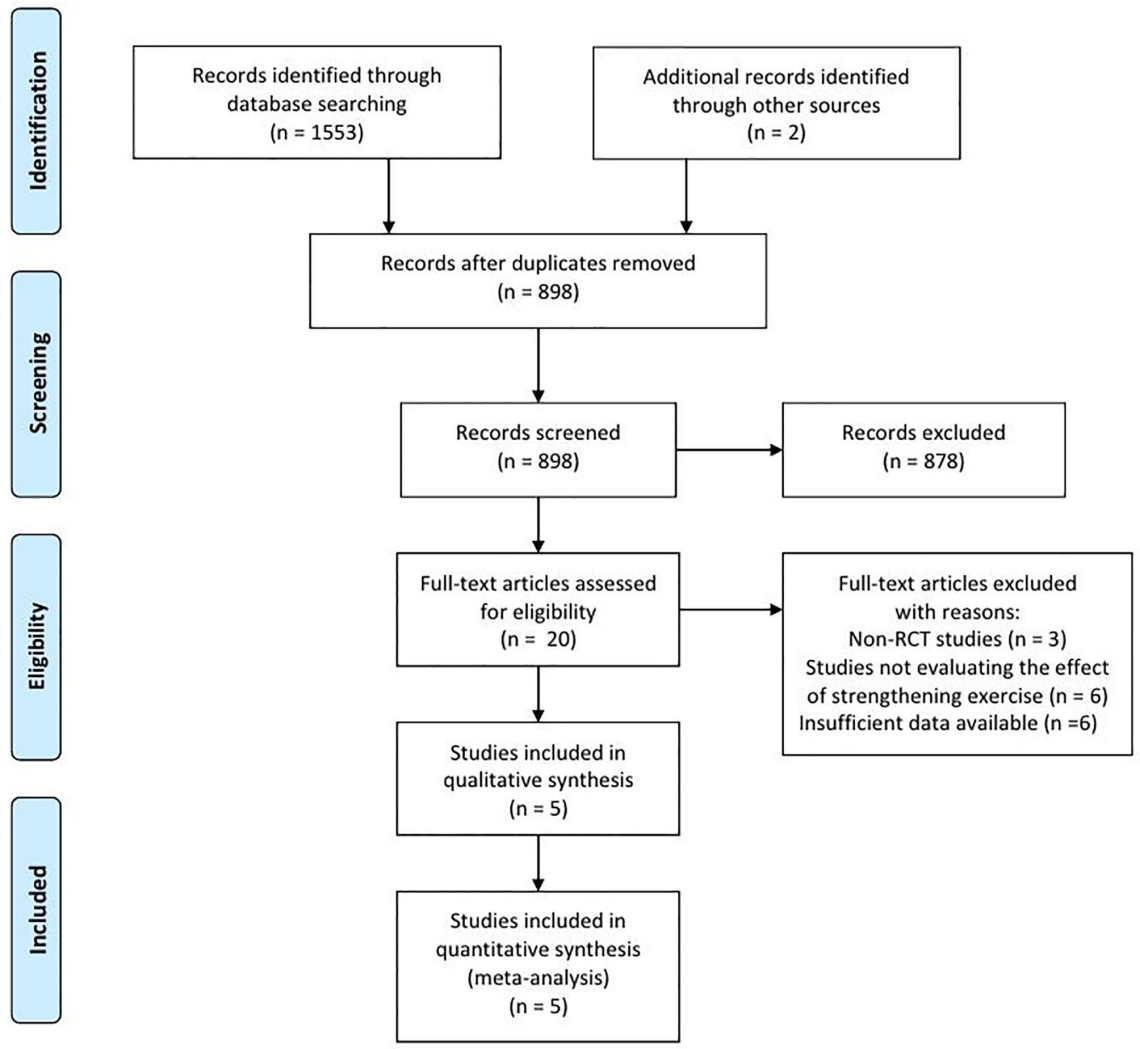
Flow diagram of study selection.

In the databases, 1,553 articles were searched, and two articles were searched by manual search. A total of 657 duplicated articles were removed (Figure 1). After screening for eligibility based on a review of the title and abstract, 20 articles were included for full-text reading. After a detailed assessment, 15 articles were excluded: three studies were not randomized controlled trials (RCT), six studies did not evaluate the effects of therapeutic exercise, and six studies reported insufficient results. Accordingly, five studies were finally included in our meta-analysis (Table 1).

**Table 1:** Characteristics of the included studies.

**References**	**Design**	**Participants (*N*, age, male %)**	**Disease duration, months**	**Exercise mode**	**Outcome assessment time, months**	**Loss to follow-up, %**	**Adverse effect (*N*)**
Drory et al. (18)	Randomized controlled trial	(25, 59.19 ± 10.53, 56.0%)	20.05 ± 0.92	Individualized trunk & limb muscle exercise (main purpose of the exercise program was to improve muscle endurance, having the muscles work against only modest loads), total 15 min, twice daily at home vs. non-exercise	3, 6, 9, and 12 months	0%	0
Bello-Haas et al., (21)	Randomized controlled trial	(27, 64.56 ± 7.28, 59.3%)	17.81 ± 9.13	Resistance exercise once-daily (individualized U/E and L/E moderate-load and moderate intensity) vs. stretching exercise	3, 6 months	22.20%	0
Braga et al. (24)	Randomized controlled trial	(48, 62.61, 67.0%)	10.80 ± 5.04	Aerobic exercise two times per week on treadmill (moderate intensity) combined with conventional rehabilitation vs. conventional rehabilitation (ROM exercises, limbs relaxation, trunk balance, and gait training)	6 month	0%	0
Kitano et al. (20)	Historical controlled trial	(105, 62.72 ± 7.91, 68.5%)	1.64 ± 1.47	Structured home-based exercise (strengthening, stretching, functional training), exercise frequency and repetitions were individualized based on patient‘s function and condition vs. usual care (without Structured home-based exercise)	6 month	5.71%	0
Zucchi et al. (25)	Randomized controlled trial	(65, 64.94, 75.38%)	16.16 ± 7.02	Intensive exercise 5 times/week (aerobic, endurance stretching or assisted active mobilization) vs. usual conventional exercise 2 times/week	3, 6, 9, 12, 18, 24 months	43.08%	5 (1 fall, 1 tracheostomy, 3 deaths)

### Study Characteristics

The five selected studies included 94 cases in experimental groups—in which the patients received therapeutic exercise—and 159 cases in the control group—in which only conventional exercise or therapy was conducted. The detailed exercise protocols are described in Table 1. Two studies (Drory et al. and Dal Bello-Haas's studies) (18, 21) used the previous version of ALSFRS for evaluating the functional statuses of the included patients, and the other three studies [Braga et al. (24), Kitano et al. (20), and Zucchi et al.'s studies (25)] used the ALS Functional Rating Scale-Revised (ALSFRS-R). While Drory et al.'s and Zicchi et al.'s study durations were 1 and 2 years, respectively, the other three studies conducted follow-ups until 6 months. For the meta-analysis, we used the 6-month follow-up data. The basic characteristics of the included studies are presented in Table 1.

### Risk of Bias

**Figure 2:**
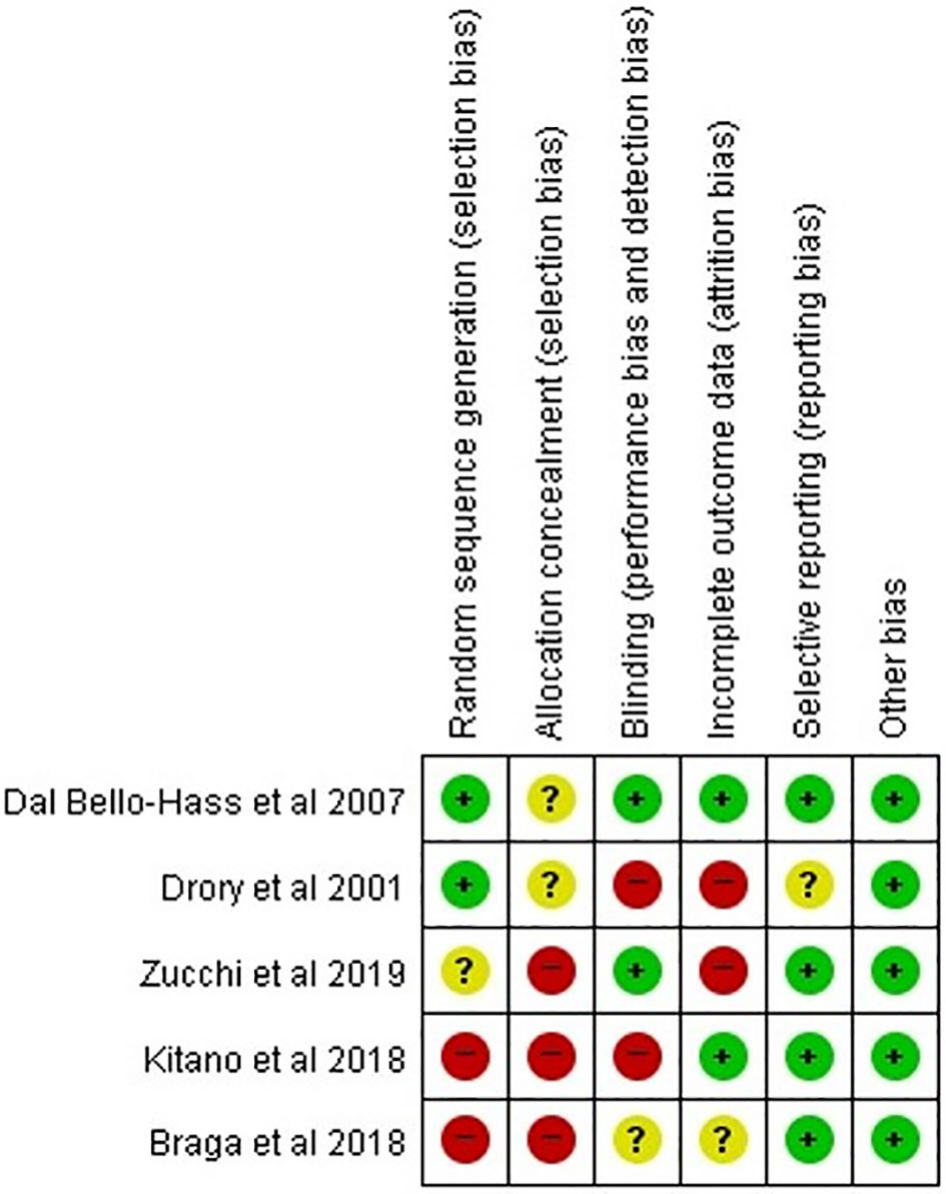
Quality assessment for selected studies.

Other than Dal Bello-Haas et al.'s study, four studies had a high or unclear risk of bias in each assessment category (Figure 2). Two studies were rated as low risk of bias in the random sequence domain. None of the studies used allocation concealment or clearly described the information on group allocation. Two studies were determined to be low risk in the domain of incomplete outcomes data (attrition bias). Regarding selective reporting, four studies (all except for Drory et al.'s study) were assessed as low risk. Of 35 domains across all studies, 15 (42.6%) were determined to be low risk. Accordingly, the overall risk of bias was assessed as high, and the studies selected for our analysis were determined to be low-quality. In the five included studies, the baseline parameters including age at onset, time from disease onset, and motor function, were adequately balanced between patients who received therapeutic exercise and those who received conventional exercise. The proportions of bulbar onset were not different between the two groups in four studies, except for Zucchi et al.'s study.

### Meta-Analysis Results

**Figure 3:**
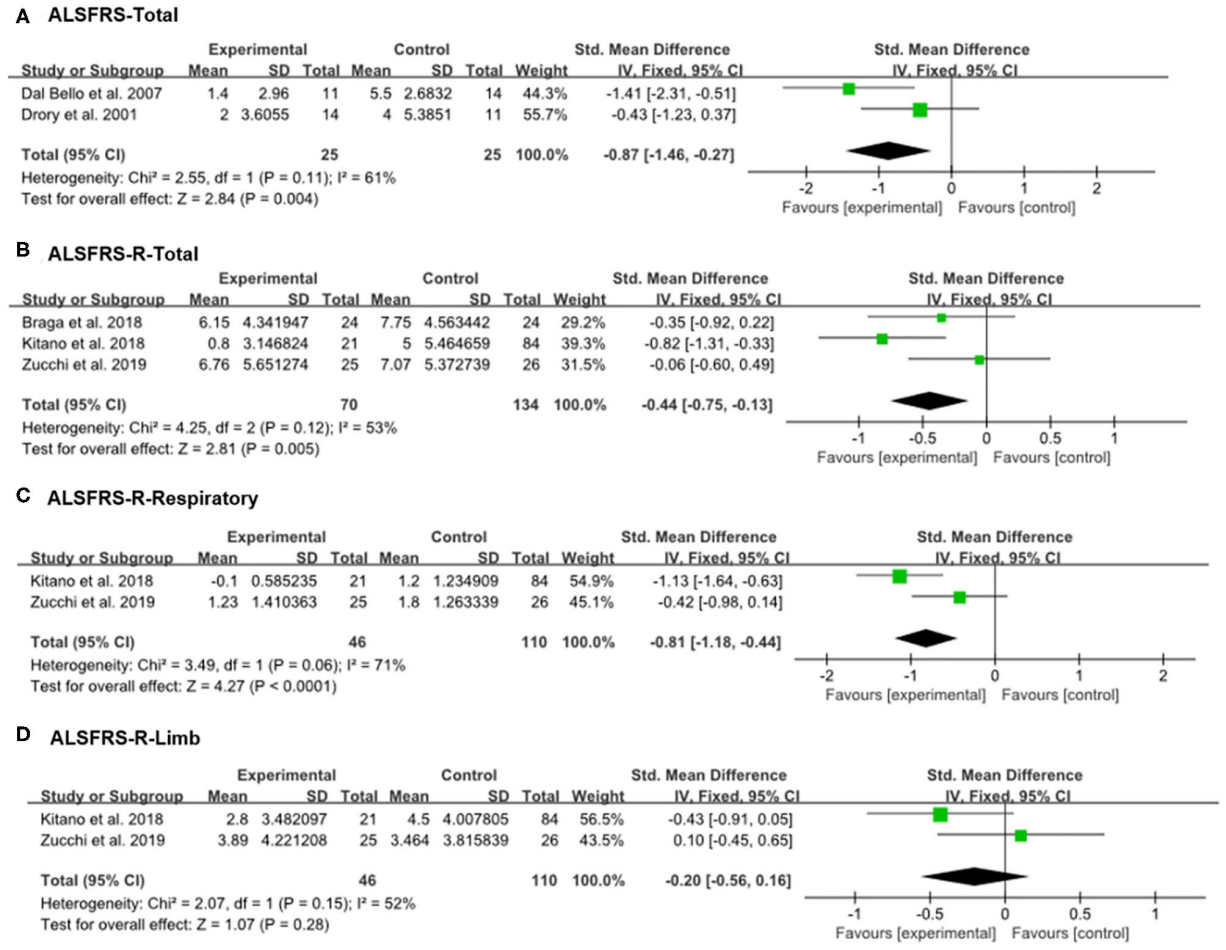
Forest plot of 6-month outcomes. **(A)** Amyotrophic lateral sclerosis functional rating scale (ALSFRS)-Total, **(B)** ALSFRS-R-Total, **(C)** ALSFRS-R-Respiratory, **(D)** ALSFRS-R-Limb.

Drory et al. and Dal Bello-Haas et al. used the previous version of the ALSFRS (18, 21). The other recent studies—including Braga et al., Kitano et al., and Zucchi et al.'s studies—assessed patients' functional status using the ALSFRS-R (20, 24, 25); therefore, we analyzed the therapeutic outcomes separately. Because the *p*-values for heterogeneity of all our analyses were >0.05, a fixed-effect model was adopted. The decrement of ALSFRS-Total scores (previous version) at the 6-month follow up was smaller in the experimental group (therapeutic exercise) than in the control group (conventional exercise or therapy) (SMD = −0.87, 95% CI = −1.46 to −0.27) (Figure 3). Likewise, the ALSFRS-R-Total scores at 6 months after initiating therapeutic exercise were less in the experimental, compared with the control group (SMD = −0.44, 95% CI = −0.75 to −0.13). In addition, the scores of ALSFRS-R-Respiratory at the 6-month evaluation were less decreased in patients who received therapeutic exercise the scores observed in the control group (SMD = −0.81, 95% CI = −1.18 to −0.44). However, the decrements of ALSFRS-R-Limb scores at the 6-month evaluation were not significantly different between the two groups (SMD = −0.20, 95% CI = −0.56 to 0.16).

### Publication Bias

**Figure 4:**
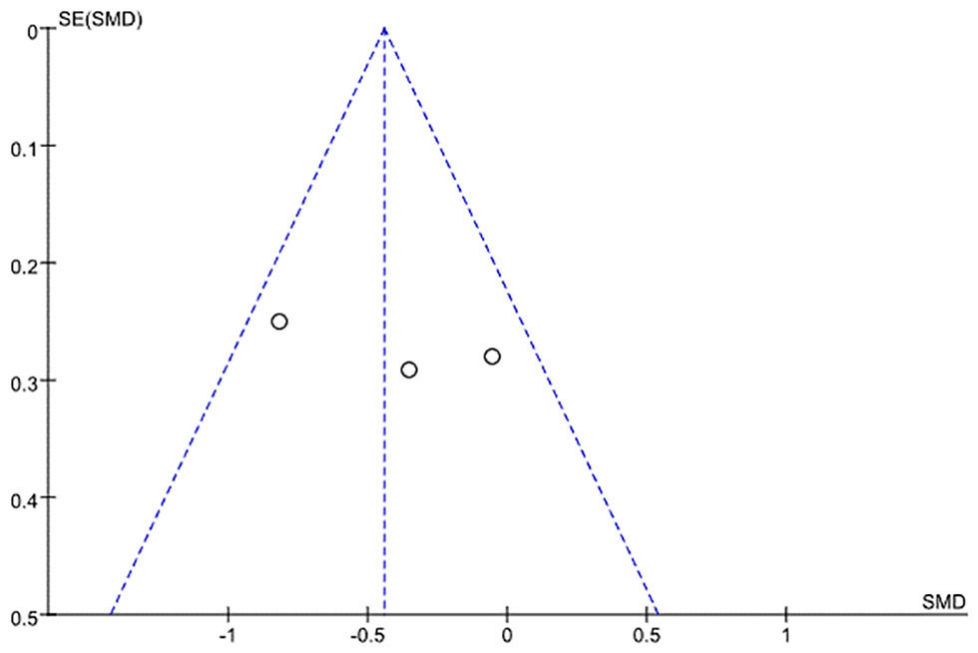
Graphic funnel plot of three studies [Braga et al. (24), Kitano et al. (20), and Zucchi et al.'s (25) studies] depicting differences of 6-month amyotrophic lateral sclerosis functional rating scale (ALSFRS)-Total score.

A funnel plot analysis and Egger's test were performed only for the ALSFRS-R-Total. The graphic funnel plot of changes in ALSFRS-R-Total scores in the three studies appeared symmetrical (Figure 4). Also, the *p*-value of Egger's test was 0.058.

## Discussion

The current meta-analysis evaluated the effectiveness of therapeutic exercise in patients with ALS. Five RCTs were finally included, and the scores of the ALSFRS or ALSFRS-R were analyzed as indicators of patients' functional statuses. The therapeutic exercise groups exhibited significantly smaller declines in ALFRS-Total or ALFRS-R-Total scores compared with the control group. The effect sized was −0.87 and −0.44 for the ALFRS-Total and ALFRS-R-Total, respectively. Based on Cohen's study (26), these outcomes mean that therapeutic exercise has a large to moderate positive therapeutic effect for slowing down the rate of functional declines in patients with ALS. Further, we analyzed the respiratory and limb functions of patients with ALS. Interestingly, only the progression of respiratory dysfunction, as measured by the ALSFORD-R-Respiratory score (effect size: −0.81), was significantly less decreased in the exercise group as compared to the control group. There was no difference in the limb function scores of the two groups, as measured by the ALSFRS-R-Limb score. Regarding these different results in the respiratory and limb motor function scores, we can suggest a possible explanation. Most of ALS patients who were included in meta-analysis study were limb-onset ALS (74.3%, 162/218) (20, 24, 25). For this reason, we think the patients participated in these studies would have weakness mainly in limb muscles, but less in respiratory muscles. Accordingly, early initiation of therapeutic exercise would have been performed primarily for respiratory muscles rather than limb muscles. It has already been reported that the early initiation of exercise in patients with ALS is more effective than starting exercise at later stages of the disease (20), therefore therapeutic exercise would be more effective for maintaining respiratory function than limb motor function in patients with ALS.

In five RCTs, adverse effects, such as increased muscle cramping, and muscle soreness or fatigue, were rarely reported by the investigators. In Zucchi 2019 (25), only one participant discontinued the study due to one of the following reasons: death, invasive ventilation, or non-invasive ventilation 23 h per day. However, in the control group, three participants discontinued the study due to at least one of these reasons. Therefore, therapeutic exercise appears to be safe for patients with ALS.

Currently, clinical management for patients with ALS is predominantly individualized (27). Although therapeutic rehabilitation—such as strengthening, resistive, or active aerobic exercises—may have a beneficial effect on the functional state of patients with ALS, it is not always included in the individualized management program. The absence of strong evidence on the efficacy of therapeutic exercise can prevent its application to patients with ALS. Appropriate exercises may be physically and psychologically beneficial to patients with ALS, especially in the earlier stages of the disease and before significant muscular atrophy or deconditioning occurs. Although exercise may not improve the strength of muscles already weakened by ALS, strengthening exercises with low to moderate weights, and aerobic exercises such as walking, swimming, and bicycling at submaximal levels (i.e., therapeutic exercise) may be important components of the overall therapeutic plan.

Our meta-analysis has some limitations. First, a limited number of trials were included. Second, due to the lack of available data for meta-analysis, various functional measures, other than ALSFRS scores, could not be analyzed. Third, the quality of the included studies was low. Fourth, as the therapeutic exercises applied in each included study were not uniform, the result of our meta-analysis should be interpreted cautiously. Fifth, this meta-analysis was not registered online. For further clarification of the effectiveness of therapeutic exercise for patients with ALS, more qualified RCTs are needed. For high quality RCTs, randomization, group concealment, and blinding of outcome measurement should be adequately performed.

## Conclusion

In conclusion, in patients with ALS, therapeutic exercise was effective in reducing the rate of declination in general physical function. Therapeutic exercise may have a particularly positive therapeutic effect on cardiopulmonary functions rather than preventing weakness in denervated or atrophy limb muscles in patients with ALS.

## Author Contributions

DP, YC, and MC: conceptualization. SK and J-SP: methodology. MC: supervision. All authors: Writing-original draft, writing-review, and editing. All authors contributed to the article and approved the submitted version.

## Conflict of Interest

The authors declare that the research was conducted in the absence of any commercial or financial relationships that could be construed as a potential conflict of interest.
